# Audiovestibular symptoms in systemic sclerosis: a systematic review and meta-analysis

**DOI:** 10.1007/s00405-024-09001-4

**Published:** 2024-10-11

**Authors:** Craig D. Salvador, Brian A. Keith, Celine Ward, Shaun A. Nguyen, Tamar Gordis, Shreya Chidarala, Emily Brennan, Habib Rizk

**Affiliations:** Medical University of South Carolina, 135 Rutledge Ave, Charleston, SC 29425 USA

**Keywords:** Scleroderma, Systemic sclerosis, Vertigo, Vestibular dysfunction, Hearing loss

## Abstract

**Purpose:**

Answer the following PICO question: Are patients diagnosed with systemic sclerosis (SSc) (Population) who are evaluated clinically and audiometrically (Intervention), have a higher prevalence of audiovestibular pathology when compared with non-SSc patients (Comparison), and how do they present symptomatically and on testing audiovestibular symptomatology and testing modalities (Outcome)?

**Methods:**

A systematic review and meta-analysis was performed. PubMed, Scopus, CINAHL, and Cochrane Library databases were searched from inception to November 27, 2023. Studies of patients diagnosed with SSc and audiologic and vestibular symptoms were selected for review. Studies of non-SSc pathologies, studies without audiovestibular outcomes, and case reports (fewer than four patients) were excluded. A meta-analysis of proportions and comparison of weighted proportions was performed in MedCalc 20.217.

**Results:**

Thirteen studies with 414 SSc patients and 390 control patients were included. The mean ± SD symptom duration was 108.5 ± 71.8 (range: 6-600) months for SSc patients. Comparison of proportions showed patients with SSc were significantly more burdened with symptoms of tinnitus (Δ34.1% [95% CI, 27.6–40.5]; *p* < 0.0001), vertigo (Δ32.4% [95% CI, 24.8–40.3]; *p* < 0.0001), and subjective hearing loss (Δ26.0% [95% CI, 20.8–31.3]; *p* < 0.0001) compared to control patients. Findings regarding vestibular testing were not meta-analyzable because of incomplete data and lack of standard reporting.

**Conclusion:**

SSc patients showed statistically significant, yet not clinically significant, worse hearing than controls. These differences, along with increased reports of subjective audiological and vestibular symptoms in patients with SSc, emphasize interdisciplinary collaboration and assessment of SSc for audiovestibular pathologies in the appropriate clinical context.

**Supplementary Information:**

The online version contains supplementary material available at 10.1007/s00405-024-09001-4.

## Introduction

With a prevalence of 50 cases per 100,000 patients and an incidence rate of approximately 5.6 per 100,000 person-years in the United States, systemic sclerosis (SSc) is a multi-organ disease process with complicated pathogenesis and symptomatology [1]. Like many autoimmune disorders, the most commonly described patient is a woman aged 30–50 years. Also known as scleroderma, SSc is characterized by inflammatory microvascular disarray, autoantibodies against cellular antigens, and diffuse fibrosis [2]. This results in vascular dysfunction, cutaneous abnormalities, and a plethora of secondary sequelae. Microvascular damage presents as an early laboratory marker of SSc, while an early clinical marker is Raynaud phenomenon [3]. While the pathophysiology is well-documented, there remains ambiguity surrounding SSc’s etiology. Some studies have found genetic markers that correlate with SSc. Polymorphisms of the OX40L gene have been associated with SSc, while the IRF5 gene has been found to correspond to both SSc and interstitial lung disease [4].

Classically, SSc has been split into two sub-categories, depending on the depth of skin involvement: limited (including morphea and linear) and diffuse (including acrosclerosis) [5]. Limited SSc (lSSc) is traditionally categorized by three phases of evolution: edematous phase, indurated phase, followed by sclerotic phase, eventually concluding as atrophic [6]. With two general subtypes, lSSc is described as morphea and linear; morphea is characterized by dermal plaques that become hypopigmented and atrophic, while linear presents as thickened and indurated skin bands, commonly in children [7]. Whereas lSSc causes cutaneous symptoms, without multi-organ involvement, diffuse SSc (dSSc) is often more severe in presentation, with various organ systems affected, and can present with pulmonary arterial hypertension (PAH), interstitial lung disease (ILD), gastrointestinal pathologies, renal dysfunction in the form of scleroderma renal crisis, heart failure, arthralgia, and myositis [8].

Consequentially, SSc patients undergo various complementary exams at annual physician appointments, such as an electrocardiogram, complete blood count, renal and liver evaluations, urinalysis, spirometry, transthoracic echocardiography, and upper gastrointestinal endoscopy [9]. Treatment regimens aim at symptom reduction and frequently involve corticosteroids, cyclophosphamide, and mycophenolate mofetil [10, 11].

Due to its variability in symptoms and evasive etiology, SSc necessitates collaboration amongst physicians across a multitude of specialties, including rheumatologists, cardiologists, gastroenterologists, pulmonologists, and nephrologists [8]. In addition, both lSSc and dSSc patients, have reported sensorineural hearing loss (SNHL), vertigo, tinnitus, and abnormalities in audiovestibular evaluations such as pure-tone audiometry (PTA), tympanograms, and stapedial reflex [12, 13]. Case reports have offered some insight, but there currently is no meta-analysis in the literature. The objective of our study was to answer the following question: Are patients diagnosed with SSc (Population) who are evaluated clinically and audiometrically (Intervention), have a higher prevalence of audiovestibular pathology when compared with non-SSc patients (Comparison), and how do they present symptomatically and on testing audiovestibular symptomatology and testing modalities (Outcome)?

## Materials and methods

### Systematic literature search

The following question was proposed: How do patients diagnosed with SSc (Population) who undergo clinical examination (Intervention), compared with non-SSc patients (Comparison), present in terms of audiovestibular symptomatology (Outcome)? A detailed search strategy (Supplemental Fig. 1) was developed with the assistance of a medical librarian in the following four databases: PubMed (National Library of Medicine, National Institutes of Health), Scopus (Elsevier), CINAHL (EBSCO), and Cochrane Library (Wiley). The search strategy used a combination of subject headings (e.g. Medical subject headings [Mesh] in PubMed). The PubMed search strategy was modified for the other three databases, replacing Mesh terms with appropriate subject headings, when available, and maintaining similar keywords. The databases were searched from inception through November 27, 2023, and results were limited to the English language. References were uploaded to Covidence systematic review software (Veritas Health Information, Melbourne, Australia) and screened for relevance.

### Selection criteria

Abstracts were first independently reviewed by four reviewers (T.M.G., S.C., C.D.S., and B.A.K.) to identify all studies pertaining to SSc patients with otologic and/or vestibular symptoms. Studies on patients with non-SSc autoimmune conditions and case reports and case series (less than four patients) were excluded. Any conflicts were resolved by discussion. To identify additional articles, the reference lists of relevant articles were hand-searched.

### Data collection

Data included in the analysis and discussion were extracted by four reviewers (T.M.G., S.C., C.D.S., and B.A.K.). Disagreements were resolved by discussion. Primary outcomes were the prevalence of subjective otologic and vestibular symptoms of SSc patients and control patients. Secondary outcomes were mean PTA and prevalences of abnormal vestibular findings in SSc and control patients. These metrics were also collected for age-matched control groups that were included in select reviews. Control groups included in these studies were patients without SSc or any other autoimmune comorbidities that were reported in the respective case series reviewed. In instances of incomplete data, an attempt was made to contact the primary author via email for clarification or sharing of previously omitted data.

### Statistical analysis

Meta-analysis of continuous measures (mean difference between controls vs. SSc patients) was performed with Cochrane Review Manager (RevMan) version 5.4 (The Cochrane Collaboration 2020). Both fixed effects and random effects models were used. This assumption is tested by the heterogeneity test or I^2^ statistic. If this test yields a low probability value (*p* < 0.05), then there is a high likelihood that fixed effects model is invalid, and the random-effects model is more appropriate. In addition, a meta-analysis of proportions was also performed using MedCalc version 22.017 (MedCalc Software Ltd, Ostend, Belgium; https://www.medcalc.org). The weighted subjective symptoms, adverse events, and serious adverse events were each expressed as a weighted proportion with 95% confidence interval (CI) given for the random effects model. Each technique was weighted according to the number of subjects treated in each study. Potential publication bias is evaluated by visual inspection of the funnel plot, which statistically examines the asymmetry of the funnel plot. In a funnel plot, the treatment effect is plotted on the horizontal axis and the standard error (SE) is plotted on the vertical axis. The vertical line represents the summary estimate derived using fixed-effects meta-analysis. Two diagonal lines represent (pseudo) 95% confidence limits (effect ± 1.96 SE) around the summary effect for each SE on the vertical axis. These show the expected distribution of studies in the absence of heterogeneity or selection bias. In the absence of heterogeneity, 95% of the studies should lie within the funnel defined by these diagonal lines. Publication bias results in the asymmetry of the funnel plot. A p-value of < 0.05 was considered to indicate a statistically significant difference for all statistical tests. Additionally, Egger’s tests with funnel plots were performed to further assess the risk of publication bias [14, 15].

### Quality of evidence assessment

Level of evidence for each selected article was evaluated with the Oxford Center for Evidence-Based Medicine. The risk of bias was assessed according to the Cochrane Handbook for Systematic Reviews of Interventions version 6.2, 2021. For nonrandomized studies, risk of bias items included the following: confounding, selection of participants into the study, classification of interventions, missing data, measurement of outcomes, and selection of reported results. Each risk of bias item was graded as low, unclear, or high. Two authors (T.M.G. and S.C.) performed a pilot assessment on three studies to check for consistency of assessment. Both then performed an independent risk assessment on the remaining studies. All disagreements were resolved by way of discussion.

## Results

### Study features

**Fig. 1:**
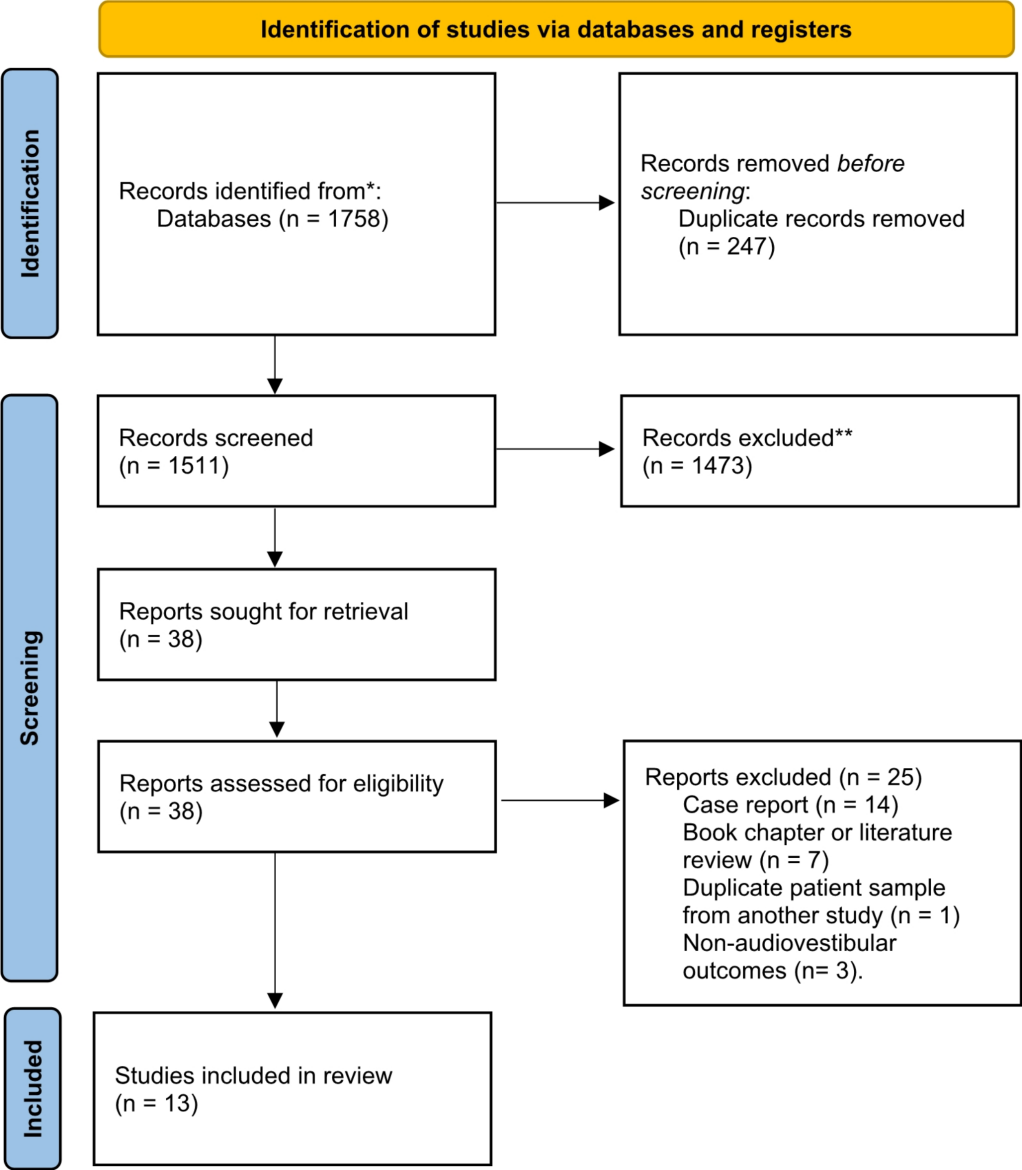
PRISMA 2020 systematic review flow diagram

**Fig. 2:**
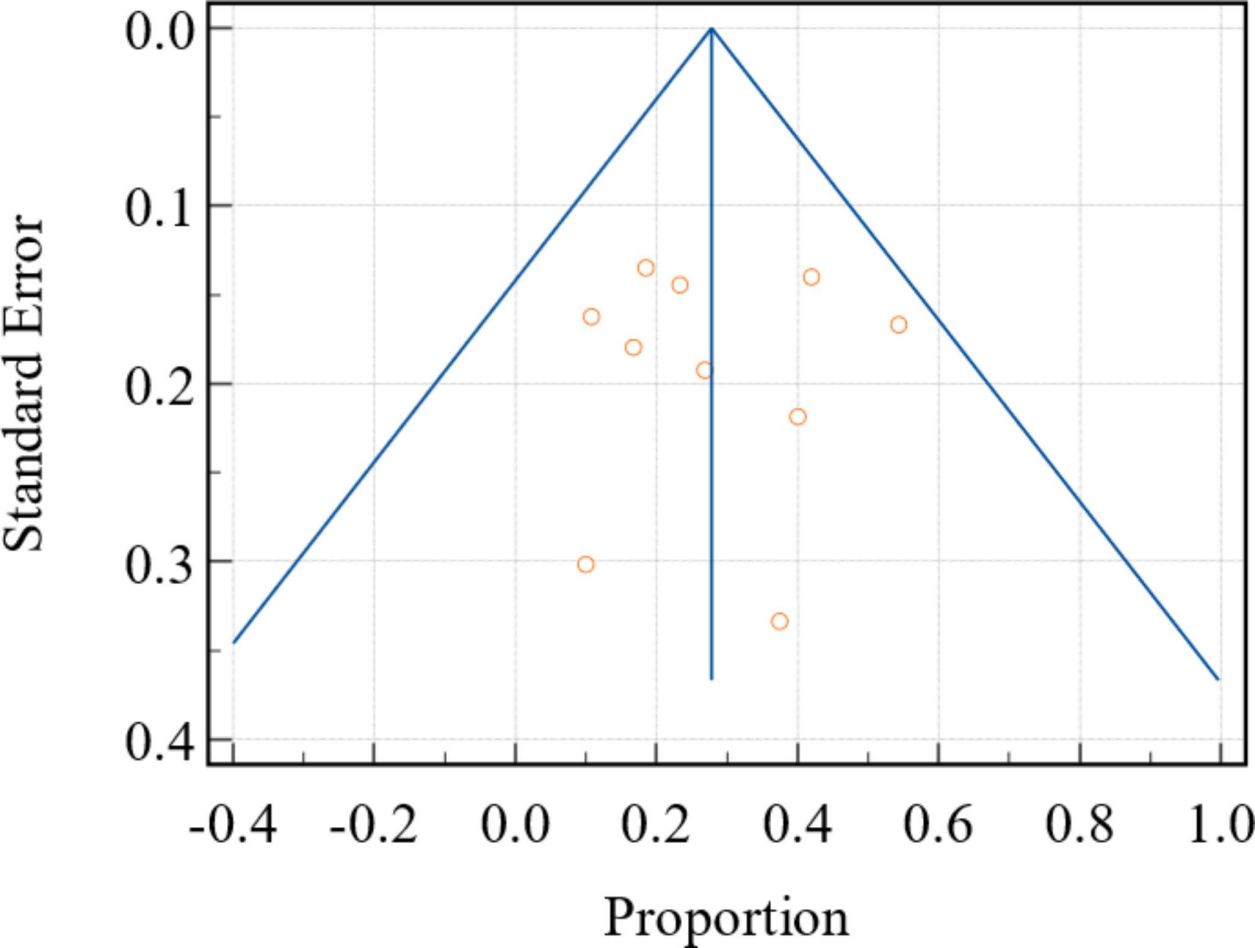
Funnel plot of meta-analysis of proportions for subjective hearing loss. This functions as a scatter plot of the studies’ observed effect sizes on the x-axis against a measure of their standard error on the y-axis; data points failing within the inverted funnel space demonstrate low publication bias

This study was conducted in accordance with the Preferred Reporting Items for Systematic Reviews and Meta-Analyses (PRISMA) guidelines (Fig. 1, Supplemental Fig. 2) [16]. A total of thirteen studies, comprised of 414 SSc patients and 390 control patients, were included [17–29]. Table 1 provides an overview of the included studies. Figure 2 shows the publication bias assessment for each of the included studies.

**Table 1:** Overview of included studies

Study	Country	Study Design	OCEBM level of evidence	Total *n*	Control *n*
Amor-Dorado 2008	Spain	Prospective cohort	II	94	59
Bassyouni 2010	Egypt	Case-control	III	59	29
Berrettini 1994	Italy	Retrospective review	III	37	0
El-Wakd 2015	Egypt	Case-control	III	60	30
Kastanioudakis 2001	Greece	Prospective cohort	II	79	45
Maciaszczyk 2011	Poland	Case-control	III	46	26
Monterio 2011	Brazil	Case-control	III	78	52
Shenavandeh 2018	Iran	Case-control	III	114	60
Silva 2019	Brazil	Prospective cohort	II	50	0
Teasdall 1980	USA	Retrospective review	III	10	0
Tsirves 2019	Greece	Case-control	III	16	8
Amor-Dorado 2023	Spain	Cross-sectional	IV	37	37
Turan 2022	Turkey	Case-control		47	44

OCEBM = Oxford Centre for Evidence-Based Medicine

### Patient demographics and symptoms

SSc patients were a mean age of 48.3 ± 10.4 (range: 19–83) years. One of the included studies reported one male in the initial cohort, but did not further categorize the gender of the 30 subjects they had final analysis on [20]. Of the available pooled data, 88.5% of our SSc cohort were female. Of the studies that reported age, control patients had a mean age of 47.3 ± 12.1 (range: 30–66) years, and out of the 293 control patients for whom sex was reported, 89.4% were female. Of the SSc patients, 68.7% (53.0-82.4, 95% CI) were classified as limited SSc, 65.4% (40.6–86.3, 95% CI) were classified as diffuse SSc, and 18.4% (6.9–33.8, 95% CI) were not reported. Meta-analysis of proportions for SSc subtypes is shown in Supplemental Table 1. The mean symptom duration was 108.5 ± 71.8 (range: 6-600) months. The most frequently reported non-audiovestibular complaints in SSc patients were Raynaud phenomenon (*n* = 210), esophageal dysmotility (*n* = 143), and telangiectasia (*n* = 114).

### Primary outcomes

Audiovestibular symptoms that were meta-analyzable consisted of subjective hearing loss, tinnitus, aural fullness, hyperacusis, dizziness, and vertigo. Meta-analysis of proportions for subjective audiovestibular symptoms in SSc patients showed that the most commonly reported symptoms were tinnitus, hyperacusis, vertigo, dizziness, subjective hearing loss, and aural fullness at **37.9%** (26.3–50.2, 95% CI), **33.9%** (23.8–45.3, 95% CI), **33.8%** (14.8–56.1, 95% CI), **32.0%** (11.3–57.4, 95% CI), **28.0%** (19.3–37.5, 95% CI), and **20.0%** (12.8–28.9, 95% CI) respectively (Table 2). Likewise, a meta-analysis of proportions was subsequently performed for subjective audiovestibular symptoms in control patients. For control patients in this study, the most commonly reported symptoms were tinnitus, subjective hearing loss, and vertigo at 3.8% (0.2–11.7, 95% CI), 2.0% (0.4–4.5, 95% CI), and 1.4% (0.2–3.4, 95% CI) respectively. In all the studies with control patients, there were no reports of subjective hyperacusis, dizziness, or aural fullness (Supplemental Table 2). Comparison of proportions showed that patients with SSc were significantly more burdened with symptoms of tinnitus at Δ34.1% ([27.6–40.5, 95% CI]; *p* < 0.0001), vertigo at Δ32.4% ([24.8–40.3, 95% CI]; *p* < 0.0001), and subjective hearing loss at Δ26.0% ([20.8–31.3, 95% CI]; *p* < 0.0001) compared to control patients. Supplemental Fig. 5 shows a forest plot of the meta-analyzed prevalence of subjective hearing loss, demonstrating [low/medium/high] publication bias amongst studies.

**Table 2:** Meta-analysis of proportions for subjective audiovestibular complaints amongst SSc patients

Study	SSc total *n*	Subjective HL *n*	Tinnitus *n*	Aural fullness *n*	Hyperacusis *n*	Dizziness *n*	Vertigo *n*
Amor-Dorado 2008	35	19	NR	NR	NR	NR	NR
Bassyouni 2010	30	NR	NR	NR	NR	NR	NR
Berrettini 1994	37	NR	NR	NR	NR	NR	NR
El-Wakd 2015	30	5	6	NR	NR	NR	18
Kastanioudakis 2001	34	NR	NR	NR	NR	NR	NR
Maciaszczyk 2011	20	8	10	6	8	7	12
Monterio 2011	26	7	5	5	NR	5	NR
Shenavandeh 2018	54	10	25	NR	NR	NR	NR
Silva 2019	50	21	32	NR	17	35	NR
Teasdall 1980	10	1	2	NR	NR	2	0
Tsirves 2019	8	3	3	0	3	NR	5
Amor-Dorado 2023	37	4	NR	NR	NR	5	4
Turan 2022	47	11	17	9	NR	NR	13
Meta-analysis of proportions [95% CI]		28.0 [19.3, 37.5]	37.9 [26.3, 50.2]	20.0 [12.8, 28.9]	33.9 [23.8, 45.3]	32.0 [11.3, 57.4]	33.8 [14.8, 56.1]

NR = not reported by study

### Secondary outcomes

**Fig. 3:**
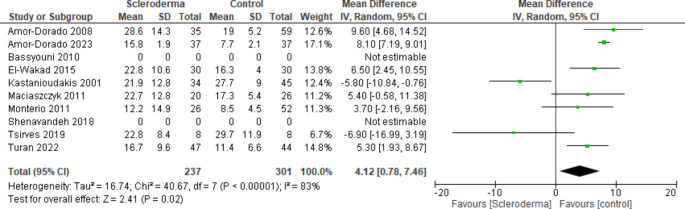
Forest plot of the mean difference between SSc patients and control patients PTA (dB). The box in the middle of each horizontal line (confidence interval, CI) represents the point estimate of the effect for a single study. The size of the box is proportional to the weight of the study in relation to the pooled estimate. The diamond represents the overall effect estimate of the meta-analysis. The placement of the center of the diamond on the x-axis represents the point estimate, and the width of the diamond represents 95% CI around the point estimate of the pooled effect

For objective otologic findings, SSc patients demonstrated a mean ± SD PTA of 21.2 ± 32.3 dB and the control group had a mean ± SD PTA of 15.3 ± 44.4 dB. The PTA mean difference between SSc patients and controls was Δ4.1 dB [0.8–7.5, 95% CI]. A forest plot for the mean difference in PTA between SSc patients and controls is shown in Fig. 3. Amongst the 88 (68 SSc, 20 controls) who were documented as undergoing stapedial reflex testing, 16.3% SSc patients and 5.1% control patients had an absent stapedial reflex. Tympanometry performed in 178 patients (104 SSc, 74 control) and reported normal type A tympanograms in 58.4% SSc patients and 41.6% control patients.

The vestibular testing modalities used by each study are outlined in Table 3. Of the thirteen studies, five described at least one method of vestibular testing in SSc patients. However, due to the low quantity of specific tests and the heterogeneity of Scleroderma subtypes between each study, the findings were not meta-analyzable. Based on the available data, the most common vestibular changes in SSc patients were in Caloric testing, positional nystagmus, the clinical test of sensory interaction on balance (CTSIB), and Dix-Hallpike [17, 19, 28]. Abnormalities in (1) caloric testing ranged from a prevalence of 0–31.4%, (2) positional nystagmus ranged from 5.4 to 60%, (3) CTSIB ranged from 24.3 to 45.7%, and (4) Dix-Hallpike ranged from 0.0 to 5.7% across all studies that reported vestibular testing [27, 30, 39]. A single study reported abnormal cVEMP findings in 80.0% of their SSc patients. Of the 80.0% of SSc patients that had abnormal cVEMP findings, 46.7% were significantly prolonged at P13 and N23 (18.5 ± 3.4 ms and 27.7 ± 2.5 ms, respectively) compared to their control cohort (12.84 ± 0.8 ms and 19.9 ± 2.5 ms, respectively) [20]. Lastly, a single study measured a significantly decreased vHIT gain in SSc patients on both sides (right: 0.91 ± 0.09; left: 0.88 ± 0.09) compared to controls (right: 0.99 ± 0.08; left 0.95 ± 0.07) [28].

**Table 3:** Descriptions of vestibular testing modalities utilized by included studies

Study	Tested *n*	Testing modality(ies) (*n* abnormal findings, %)	Further details
Amor-Dorado 2008	35	Caloric (11, 31.4) Cephalic rotation (3, 8.6) CTSIB (16, 45.7) Dix-Hallpike (2, 5.7) Evoked nystagmus (0, 0.0) Oculographic test (5, 14.3) Positional nystagmus (21, 60.0) Spontaneous nystagmus (2, 5.7)	
Bassyouni 2011	NR	Sensory organization of CDP on EquiTest*	Median of 66.5, range of 19.0–78.0
Berrettini 1994	37	Caloric (1, 2.7) Positional nystagmus (2, 5.4)	
El-Wakd 2015	30	cVEMP (14, 46.7)	Absent cVEMP = 6, delayed cVEMP = 14, mixed absent/delayed cVEMP = 4
Kastanioudakis 2001	NR	NR	No vestibular testing performed/described
Maciaszczyk 2011	NR	NR	No vestibular testing performed/described
Monterio 2011	NR	NR	No vestibular testing performed on 2 patients: reasons unspecified
Shenavandeh 2018	NR	NR	No vestibular testing performed/described
Silva 2019	NR	NR	No vestibular testing performed/described
Teasdall 1980	NR	NR	No vestibular testing performed/described
Tsirves 2019	NR	NR	No vestibular testing performed on 5 patients: reasons unspecified
Amor-Dorado 2023	37	Spontaneous nystagmus (0, 0.0) Gaze-evoked nystagmus (0, 0.0) OCR (0, 0.0) Positional nystagmus (2, 5.4) Dix-Hallpike (0, 0.0) vHIT Head-shaking test (0, 0.0) Caloric testing (0, 0.0) CTSIB (9, 24.3) CDP (9, 24.3)	No VEMP testing was performed: multiple sites did not have available equipment to perform this test. vHIT Right Gain, mean (SD) = 0.91 (0.09) vHIT Left Gain, mean (SD) = 0.88 (0.09)
Turan 2022	NR	NR	No vestibular testing performed/described

CDP = Computerized Dynamic Posturography

CTSIB = Clinical Test of Sensory Interaction on Balance

cVEMP = Cervical Vestibular Evoked Myognic Potential

OCR = Oculocephalic response

vHIT = Video Head Impulse Test

NR = not reported

*Median vestibular ratio is the vestibular changing condition over the control condition

## Discussion

Previous studies have tried to assess the prevalence of audiovestibular symptoms in patients with SSc. However, these studies are scarce and furthermore, the available studies are limited by small sample sizes. To date, this study is the first known meta-analysis of audiovestibular symptom prevalence in patients with SSc compared to non-SSc patients. In our study, we found that patients with SSc presented with significantly increased rates of audiovestibular symptoms, thus highlighting the need for close monitoring and follow-up by appropriate specialists.

In this meta-analysis, we found that patients with SSc presented with tinnitus at a rate of 34.1%, vertigo at 32.4%, and subjective hearing loss at 20.8% higher than non-SSc patients. Additionally, out of our study sample, only patients with SSc were reported to have complaints of hyperacusis and aural fullness (Table 2). This prevalence data shows that patients with SSc are significantly more burdened by audiovestibular symptoms than those without SSc, with the prevalence of SSc patients developing any audiovestibular symptom being 1 in every 5 SSc patients on a lower estimation, to 1 in every 3 cases of SSc on higher estimations. While this study is the first known meta-analysis of the prevalence of audiovestibular symptoms in SSc, our prevalence data aligns well with a previous systematic review that found a range of prevalence for hearing loss from 20 to 77% and a range of prevalence of vestibular disorders from 11 to 63% in patients with SSc [30].

From a vestibular standpoint, vestibular changes in SSc patients were more difficult to characterize due to the heterogeneity in vestibular testing modalities across all included studies, therefore, a meta-analysis could not be performed on the prevalence of objective vestibular abnormalities in SSc patients. Yet from the available data, we found that abnormal vestibular testing manifested in a wide range of prevalences from 24.3 to 45.7% using CTSIB and 2.7–31.4% using caloric testing [17, 19, 20, 28]. The wide range of prevalences observed can largely be explained by the lack of standardized testing modalities for exploring vestibulopathies at the time of publication of each study. For example, the two studies by Amor-Dorado et al. (2008 and 2023) employed several comprehensive vestibular tests such as CTSIB and vHIT while the study by El-Wakd (2015) utilized only cVEMP testing [17, 20, 28]. Other included studies only assessed the presence of nystagmus which is less specific for isolating the presence of vestibular dysfunction.

Although we present evidence that audiovestibular symptoms are more prevalent in SSc patients compared to non-SSc patients, the underlying pathophysiology of inner ear dysfunction in SSc remains unclear. From an audiological standpoint, inner ear dysfunction in systemic autoimmune diseases has been clinically correlated and well-documented, especially in vascular-compromising diseases [31, 32]. Early onset microvascular damage through obliterative microvascular lesions and the rarefaction of capillaries are hallmarks of SSc, followed by fibrosis that then disrupts healthy tissue [3, 33]. More specifically, the cochlea is highly sensitive to oxidative changes, which is central to the pathogenesis of SSc. Although rare, the natural vasculopathy in SSc may also be compounded by comorbid ANCA-associated vasculitis, a condition associated with a more severe SSc disease course [34, 35]. The predominant consensus is that poor perfusion leads to poor nutrient delivery and build-up of oxidative stress, causing hair cell damage [36, 37]. Clinically, this manifests itself as SNHL, which the majority of our cohort of SSc patients who had PTA testing developed. A more recent study corroborates this leading theory as investigators show that blood vessels of the lower basal turn of the cochlea were occluded and that vessels in the apical turn of the cochlea were inflamed in SSc patients compared to age-matched controls. Thus, they conclude that vasculitic and fibrotic processes of the stria vascularis are the leading lesions that lead to audiologic impairment in patients with SSc [38]. Additionally, SSc patients may present with audiologic symptoms at increased rates as a part of a side effect profile from being systemic immunosuppressants for the management of their disease. Management of SSc is dependent on the affected organ system, however, systemic immunosuppression is generally recommended during episodes of worsened symptoms with medications including mycophenolate mofetil and methotrexate [39, 40]. A recent systematic review found that 97.7% of their cohort of patients developed bilateral SNHL and 39.7% developed tinnitus after therapy with medications such as mycophenolate mofetil, methotrexate, and calcineurin inhibitors [41]. In this study, we found that about a third of SSc patients will develop symptoms of vertigo. Yet, from a vestibular standpoint, the exact mechanism of how SSc impairs vestibular function is not fully understood and remains highly speculative. Several investigators and authors have characterized potential mechanisms of vestibular pathology in other systemic autoimmune syndromes. Bovo et al. posit that the leading theory for vestibular impairment in patients with systemic autoimmune syndromes is likely from cross-reaction from antibodies or rogue T cells that cause unintentional inner ear damage [32]. Rahne et al. reported on vasculitic processes and concluded that vestibular dysfunction is likely due to temporary occlusion of the labyrinthine and/or anterior vestibular artery [42]. One of our included studies found greater vestibular impairment in SSc patients with increased skin thickness, thus concluding that collagen buildup in the ECM disrupts the interstitial fluid pressure, which is key for endolymphatic outflow [17, 43]. Although less likely, medication side effects could play a role in SSc patients presenting with increased rates of complaints such as vertigo or dizziness (1 out of every 3 SSc patients). In the same systematic review cited above, the authors reported a low prevalence of complaints of vertigo in patients taking systemic immunosuppressants at a rate of 0.02% [41].

Despite the evidence that patients with SSc are significantly more burdened with audiovestibular symptoms, our meta-analysis reveals that patients with SSc presented with a mean PTA of 4.1 [95% CI, 0.8–7.5] dB higher than non-SSc patients. While this mean difference is statistically significant, this difference is likely not clinically significant. A difference in PTA of 4.1 dB is quite small and does not necessarily imply the need for audiological evaluation beyond what is recommended to the general population. According to the Academy of Audiology, a hearing loss of up to 20 dB below the threshold is considered normal hearing and a loss of 20–40 dB is mild [44]. However, in our study sample, patients with SSc had a pooled mean (SD) PTA of 21.2 (32.2) dB, thus barely meeting the cutoff for the category of having mild hearing loss. However, given the mean age of patients with SSc in this meta-analysis being 48.3 years of age, it is challenging to determine if the mild hearing loss observed may be due to age-related deprivation of hearing function as opposed to disease processes of SSc.

In this study, we demonstrate evidence that patients with SSc present with audiovestibular symptoms at higher prevalences compared to non-SSc patients. As such, these findings carry with them tremendous negative implications for the quality of life of affected patients. Specifically, mild hearing loss can still profoundly affect patients’ lives. Newman et al. administered the Hearing Handicap Inventory for Adults (HHIA) to patients with mild hearing loss and found that patients reported a variety of emotional and social-situational problems [45]. A meta-analysis found that the treatment of tinnitus with either a hearing aid or a sound generator was associated with a clinically significant reduction in tinnitus symptom severity scores, which is a measure of tinnitus-specific quality of life and symptom burden [46]. Similarly, Gamiz et al. assessed vertigo patients with the Medical Outcomes Study 36-Item Short Form Survey (SF-36) and the Dizziness Handicap Inventory Survey Short Form (DHI-S), reporting that patients who suffered from vertigo had significantly increased severity of body pain, social function, and mental health scores in comparison with the general population [47]. Outside of quality-of-life, untreated vertigo, and other vestibular impairments have been linked to increased morbidity and mortality, especially in the aging population, due to fall-trauma-related injuries and complications [48]. While, our analysis revealed the mean age of SSc patients with audiovestibular symptoms to be in the fourth or fifth decade of life, at 48.3 ± 10.4 years, a high prevalence of SSc patients with vestibular complaints may suffer in the long term from under-recognized, and thus poorly managed fall-trauma-related morbidity and mortality. Therefore, while additional audiovestibular testing is not typically warranted for patients diagnosed with SSc, this population would see great benefit to the quality of life and overall safety, with additional assessment, should audiovestibular symptoms arise. Furthermore, vertigo may be an underestimated manifestation of autoimmune diseases, and vice versa, sudden onset of vertigo may be the presenting symptoms of an undiagnosed autoimmune disorder, thus highlighting the strong need for and importance of a multidisciplinary approach to care for SSc patients [49]. Thus, physicians assessing patients with a systemic autoimmune disease should be wary of potential audiovestibular dysfunction, and likewise, Otolaryngologists assessing sudden onset audiovestibular symptoms should be wary of potential underlying systemic autoimmune processes.

One of the biggest limitations of this study was the sample size. While thirteen studies met the inclusion criteria, ten of them included control patients for comparison, and of these, only eight provided primary outcomes for SSc and control patients [17, 20, 22–24, 27–30]. Without additional studies to raise the sample size, the power of the study is relatively low. This is echoed by the high risk of bias in the selection of reported results that were found for nearly 50% of the included studies. By reporting the primary outcome metrics in more patients, the sample size can increase and raise the study’s power. Furthermore, by sharing all the information indicated in the methods section in their results and discussion, study authors will help to lower the risk of bias. Another potential limitation of the study regarding the primary outcomes are the potential ambiguity in the classification of subjective symptoms. Quite often, dizziness and vertigo are used interchangeably in the colloquial diction, although for otolaryngologists, vertigo indicates an illusion of movement (spinning or non-spinning) and often but not always correlates with peripheral vestibulopathy, whereas dizziness is an illusion of spatial disorientation that can occur in peripheral or central vestibulopathy as well as non-vestibular pathologies including hemodynamic pathologies [50]. Thus, future studies should look at the symptoms in more detail and correlate them to vestibular testing results and finalized diagnoses of these complaints. Lastly, while the meta-analysis of prevalence data is a critical field of study, the clinical utility of its findings are under debate. There is a lack of clear guidelines on how to interpret meta-analysis of prevalences which may limit the quality of our methods and the conclusions we can draw from audiovestibular symptom prevalence in patients in SSc versus patients without SSc. Moreover, our meta-analysis on audiovestibular symptom prevalence was on subjective complaints. The subjectivity of the prevalence data may also add additional limits to the conclusions that can be drawn from the pooled data. Studies examining audiovestibular symptoms are inherently limited in their ability to measure all symptoms objectively, for example, tinnitus, dizziness, and vertigo severity. However, future studies can quantize audiovestibular symptom severity by utilizing patient-reported outcome metrics. These metrics would allow researchers to directly compare symptom severity between patients with SSc and those without SSc.

## Conclusion

SSc is associated with various secondary, multi-organ pathologies that warrant additional annual evaluations and tailored treatments. While pulmonary, gastrointestinal, and cardiologic sequelae have been well documented, otologic and vestibular sequelae remain more elusive. In this study, we demonstrate that patients with SSc have significantly higher prevalences of audiovestibular symptoms compared to patients without SSc. Additionally, we show that patients with SSc have statistically, albeit not clinically, significantly worse PTA values than non-SSc patients. While these findings do not indicate the need for additional audiometric and vestibular testing in SSc patients, they emphasize the need to appropriately evaluate these prevalent complaints which could lead to quality-of-life improvement Despite the higher prevalence of vestibular symptoms in SSc identified in our study, the mechanism has not yet been fully elucidated. We believe this knowledge gap could be resolved by future studies exploring the mechanisms behind vestibular impairment in systemic autoimmune diseases such as SSc and with additional clinical studies looking to characterize the nature of the vestibular complaint and correlate it with testing or other additional investigations.

## Electronic Supplementary Material

Below is the link to the electronic supplementary material.

Supplementary Material 1

Supplementary Material 2

Supplementary Material 3

Supplementary Material 4

Supplementary Material 5
