# Supplementary material for: Audiovestibular symptoms in systemic sclerosis: a systematic review and meta-analysis
Source: Eur Arch Otorhinolaryngol. 2024 Oct 11;282(3):1147–57. doi: 10.1007/s00405-024-09001-4 (PMC11890250; doi:10.1007/s00405-024-09001-4)
Supplement: Supplementary file 1 — Supplementary Material 1 [file 405_2024_9001_MOESM1_ESM.docx]

**Supplemental Figure 1.** Detailed search terms for each database

November 27, 2023

PubMed

| ("scleroderma"[all fields] OR "diffuse" sclerosis[all fields] OR "skin thickening"[all fields] OR "excessive collagen deposition" [all fields] OR "fibrosing disease" [all fields] OR "morphea"[all fields] OR "systemic sclerosis"[all fields] OR "SSc"[all fields] OR "CREST" [all fields] OR "Scleroderma, Systemic"[Mesh] OR "Scleroderma, Localized"[Mesh] OR "Scleroderma, Limited"[Mesh] OR "Scleroderma, Diffuse"[Mesh]) OR "CREST Syndrome"[Mesh]) AND ("ENT"[all fields] OR "otolaryngology" [all fields] OR "otology"[all fields] OR "hearing" loss [all fields] OR "deafness" [all fields] OR "tinnitus" [all fields] OR "vertigo" [all fields] OR "dizziness" [all fields] OR "sensorineural" [all fields] OR "conductive" [all fields] OR “auditory”[all fields] OR “vestibul*” [all fields] OR labyrin* [all fields] OR "Neurotology"[Mesh] OR "Hearing Loss"[Mesh] OR "Tinnitus"[Mesh] OR "Vertigo"[Mesh] OR "Dizziness"[Mesh] OR "Deafness"[Mesh] OR "Vestibular Diseases"[Mesh] OR "Auditory Diseases, Central"[Mesh] OR "Hair Cells, Auditory, Inner"[Mesh]) | English filtered: 1022 |
| --- | --- |

November 27, 2023

Scopus

| TITLE-ABS-KEY (( scleroderm* OR lipodermatosclerosis OR {diffuse sclerosis} OR {skin thickening} OR {stiff skin} OR {excessive collagen deposition} OR {fibrosing disease} OR morphea OR morphoea OR {systemic sclerosis} OR ssc OR {CREST syndrome} ) AND ( auditory OR conductive OR deaf* OR dizzy OR dizziness OR ent OR hearing OR labyrin* OR neurotology OR otolaryngology OR otology OR sensorineural OR tinnitus OR vertigo OR vestibul* )) | English filtered: 688 |
| --- | --- |

November 27, 2023

CINAHL

| (MH "Scleroderma, Systemic+" OR MH "Scleroderma, Limited" OR MH "Scleroderma, Circumscribed" OR MH "CREST Syndrome" OR scleroderm* OR lipodermatosclerosis OR “diffuse sclerosis” OR "skin thickening" OR “stiff skin” OR "excessive collagen deposition" OR "fibrosing disease"[tiab] OR morphea[tiab] OR morphoea[tiab] OR "systemic sclerosis”[tiab] OR SSc[tiab] OR “CREST syndrome”) **AND** (MH "Auditory Diseases, Central" OR auditory OR conductive OR MH "Deafness+" OR deaf* OR MH "Dizziness" OR dizzy OR dizziness OR ENT OR MH "Hearing Loss, Functional" OR hearing OR labyrin* OR neurotology OR otolaryngology OR otology OR sensorineural OR MH "Tinnitus" OR tinnitus OR MH "Vertigo+" OR vertigo OR MH "Vestibular Diseases+" OR vestibul*) | English filtered: 41 |
| --- | --- |

November 27, 2023

Cochrane

| ("systemic scleroderma" OR "diffuse scleroderma" OR "limited scleroderma" OR "CREST Syndrome" OR "localized scleroderma" OR scleroderm* OR lipodermatosclerosis OR "diffuse sclerosis" OR "skin thickening" OR "stiff skin" OR "excessive collagen deposition" OR "fibrosing disease" OR morphea OR morphoea OR "systemic sclerosis" OR SSc OR "CREST syndrome" OR CREST) AND ("central auditory diseases" OR auditory OR conductive OR "deafness" OR deaf* OR "dizziness" OR dizzy OR dizziness OR ENT OR "inner hair cells" OR "hearing loss" OR hearing OR labyrin* OR "Neurotology" OR neurotology OR otolaryngology OR otology OR sensorineural OR "tinnitus" OR tinnitus OR "vertigo" OR vertigo OR "vestibular diseases" OR vestibul*) | English filtered: 7 |
| --- | --- |
