# Supplementary figures and images for: Audiovestibular symptoms in systemic sclerosis: a systematic review and meta-analysis
Source: Eur Arch Otorhinolaryngol. 2024 Oct 11;282(3):1147–57. doi: 10.1007/s00405-024-09001-4 (PMC11890250; doi:10.1007/s00405-024-09001-4)

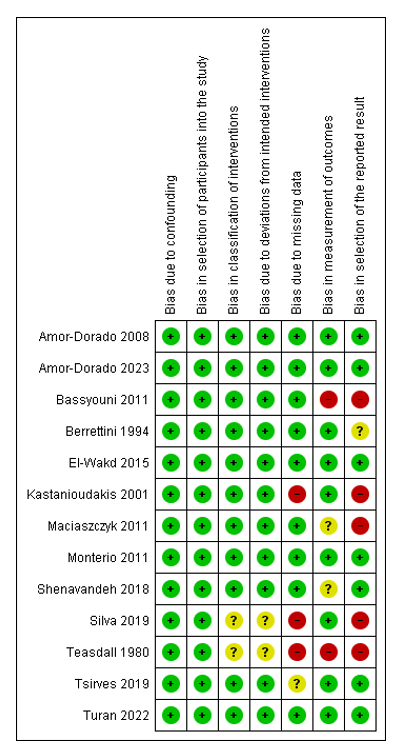

Supplement: Supplementary file 3 — Supplementary Material 3 [file 405_2024_9001_MOESM3_ESM.tiff]

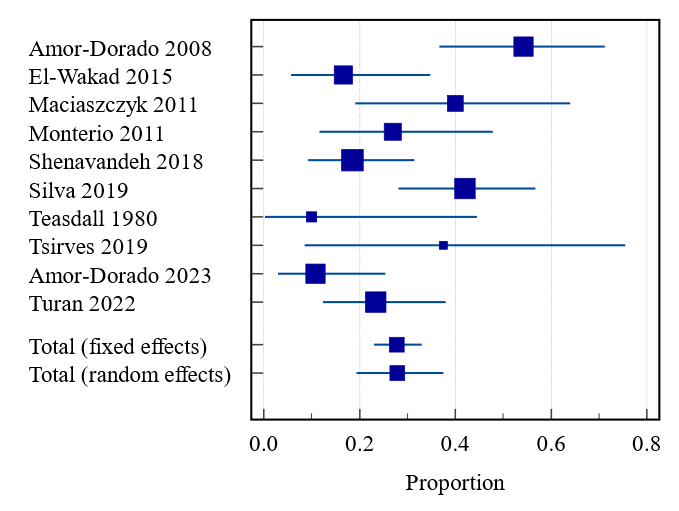

Supplement: Supplementary file 4 — Supplementary Material 4 [file 405_2024_9001_MOESM4_ESM.tiff]
