# Supplementary material for: Audiovestibular symptoms in systemic sclerosis: a systematic review and meta-analysis
Source: Eur Arch Otorhinolaryngol. 2024 Oct 11;282(3):1147–57. doi: 10.1007/s00405-024-09001-4 (PMC11890250; doi:10.1007/s00405-024-09001-4)
Supplement: Supplementary file 5 — Supplementary Material 5 [file 405_2024_9001_MOESM5_ESM.docx]

**Supplemental Figure 1.** Detailed search terms for each database

**Supplemental Figure 2.** PRISMA 2020 checklist

**Supplemental Figure 3.** Risk of bias assessment

**Supplemental Figure 4.** Forest plot of meta-analysis of proportions for subjective hearing loss. The box in the middle of each horizontal line (confidence interval, CI) represents the point of estimate of the effect for a single study. The size of the box is proportional to the weight of the study in relation to the pooled estimate. The diamond represents the overall effect estimate of the meta-analysis. The placement of the center of the diamond on the x-axis represents the point estimate, and the width of the diamond represents the 95% CI around the point estimate of the pooled effect.
